# Supplementary material for: Plasticity-Enhancing Effects of Levodopa Treatment after Stroke
Source: Int J Mol Sci. 2021 Sep 23;22(19):10226. doi: 10.3390/ijms221910226 (PMC8508853; doi:10.3390/ijms221910226)
Supplement: Supplementary file 1 [file ijms-22-10226-s001.zip › ijms-1335292-supplementary.pdf]

SUPPLEMENTAL MATERIAL

Plasticity enhancing effects of levodopa treatment after stroke

Daniela Talhada, PhD,[1] Niklas Marklund, MD, PhD,[2] Tadeusz Wieloch, PhD,[1] Enida Kuric, PhD,[1] Karsten Ruscher, MD[1,2*]

[1]Laboratory for Experimental Brain Research, Division of Neurosurgery, Department of Clinical Sciences, Lund University, Sweden.

[2]LUBIN Lab - Lunds Laboratorium för Neurokirurgisk Hjärnskadeforskning, Division of Neurosurgery, Department of Clinical Sciences, Lund University, Sweden.

*Corresponding Author: Dr. Karsten Ruscher, Associate Professor, Laboratory for Experimental Brain Research, Wallenberg Neuroscience Center, Lund University, BMC A13, S-22184 Lund, Sweden. Tel +46-46-2220613. email: karsten.ruscher@med.lu.se

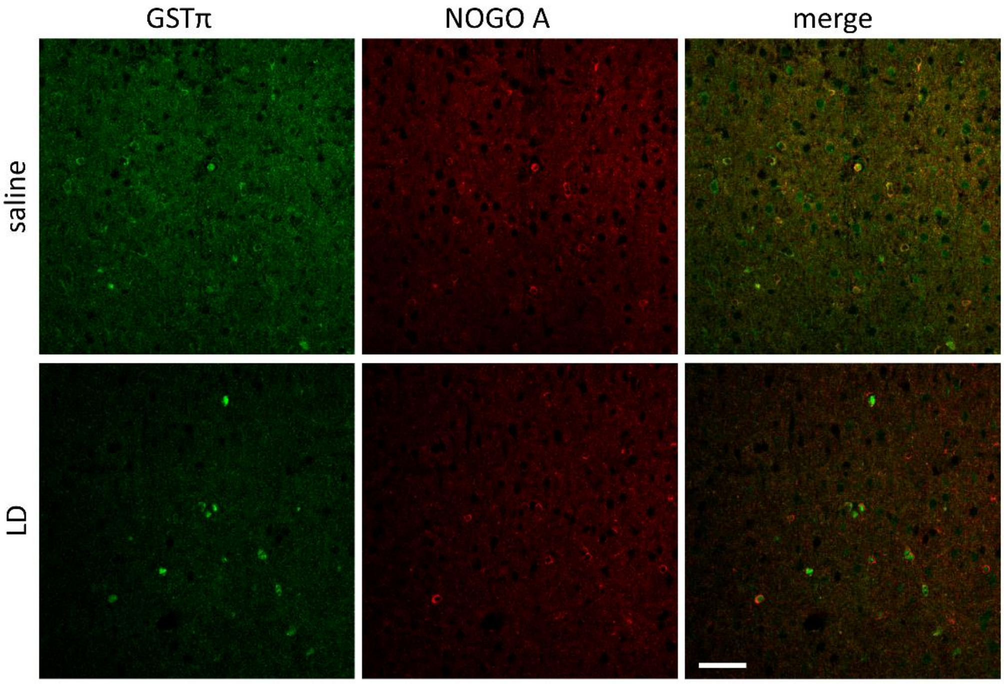

Supplemental Figure I. Representative co-staining for glutathione-S-transferase π (GST-π, green, AF488) and Nogo A (red, Cy3) in the homotypic brain region of Sham operated rats, correspondent to the cortical peri infarct area of stroke animals. No statistical difference was observed in the quantification of NogoA+ oligodendrocytes between rats treated with saline (vehicle) or levodopa (LD 20 mg/kg) 14 days after transient middle cerebral artery occlusion. Scale bar 50 µm.
